# Influenza B virus from respiratory samples of individuals with influenza-like illness in a University Campus in Arizona USA, in February 2025

**DOI:** 10.1128/mra.00344-25

**Published:** 2025-07-07

**Authors:** Angelica U. Negrete, Stacy Nelson, Matthew Scotch, Temitope O. C. Faleye

**Affiliations:** 1 The Biodesign Institute Center for Environmental Health Engineering, Arizona State University 7864, Tempe, Arizona, USA; 2 ASU Health Services, Arizona State University, Tempe, Arizona, USA; 3 College of Health Solutions, Arizona State University, Phoenix, Arizona, USA; Queens College Department of Biology, Queens, New York, USA

**Keywords:** influenza B virus, Arizona, flu-like illness, B/Victoria lineage

## Abstract

We describe three influenza B viruses detected in respiratory samples from individuals with flu-like symptoms on a university campus in Arizona, USA, in February 2025. Analysis of the hemagglutinin segments shows all three are B/Victoria, clade V1A.3a.2, and subclade C.5.1 with NA segments that belong to clade B.7.

## ANNOUNCEMENT

Influenza viruses are members of the family *Orthomyxoviridae*. Members of four of the genera (influenza A virus [IAV], IBV, ICV, and IDV) in the family have been associated with respiratory infections in humans and animals. The enveloped, pleomorphic virions usually encase eight-segment, negative-sense RNA genomes of about 14 kb (1). For prevention and control, the hemagglutinin (HA) and neuraminidase (NA) segments of IBV are of utmost importance. Two IBV lineages (Victoria and Yamagata; based on HA) have traditionally been documented to circulate mainly in humans (2). Yamagata has not been confirmed since March 2020, leading to speculation that it may be extinct (3). Here, we describe three IBV variants detected within a week in February 2025, on a university campus in Tempe, Arizona, USA.

The nasal swabs analyzed here were collected within a week (10th, 12th, and 13th) in February 2025 as part of an ongoing influenza-like illness surveillance program on a university campus in Arizona, USA. Samples were flagged as containing IBV using the rapid OneStep + Ultra Influenza A and B antigen detection test kit (Henry Schein, Melville, New York, USA) as part of routine clinical care at the university health services. Subsequently, the samples were transported to our laboratory where RNA was extracted from the sample using QIAamp viral RNA minikit (ThermoFisher Scientific, Waltham, MA, USA) following the manufacturer’s instructions. The extract was used for cDNA synthesis using the SSIV first-strand cDNA synthesis kit (ThermoFisher Scientific, Waltham, MA, USA) and BHANAF primer (4). The HA, NA, and nonstructural (NS) segments were amplified using the BHANAF and BHANAR primers (4) alongside Phusion-plus PCR master mix (ThermoFisher Scientific, Waltham, MA, USA) as previously described (4, 5). Amplicons were cleaned using magnetic beads (Quantabio, Beverly, MA, USA). Sequencing libraries were prepared using the ligation sequencing amplicons Native Barcoding Kit 24 V14 (SQK-NBD114.24), pooled, and sequenced on a Flongle flow cell and MK1b. Base calling, trimming, and template-guided assembly were done using MinKNOW v24.11.10, Porechop v0.2.4 (6), and Minimap v2.24 (7), respectively. IBV contigs were typed using both a BLASTn search of the GenBank database and Nextclade (8). Trees generated from Nextclade were annotated using Figtree v1.4.4. Default parameters were used for all software except where otherwise noted.

**Fig 1:**
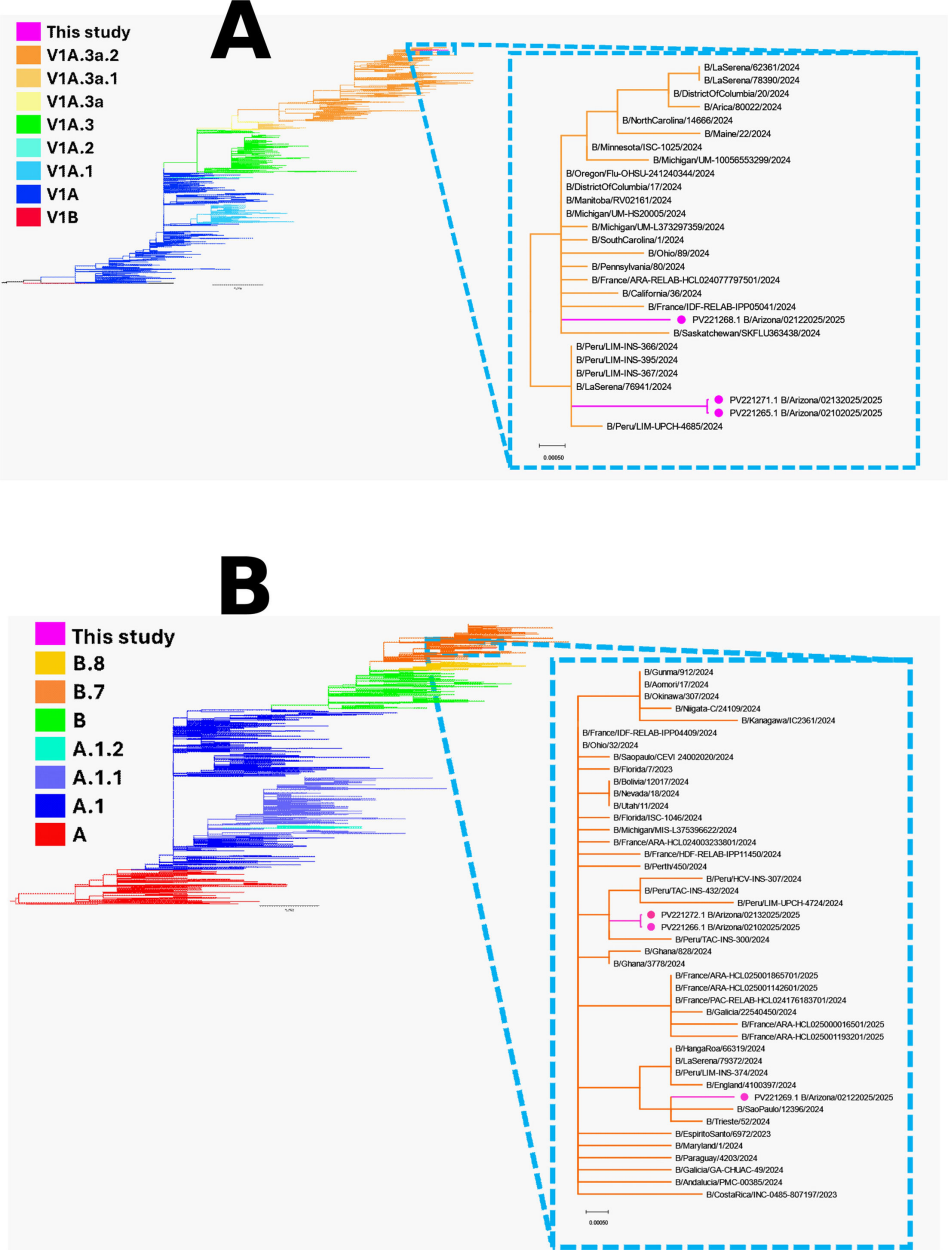
Phylogenetic trees showing influenza B virus HA (**A**) and NA (**B**) detected in this study (highlighted using purple circles). The trees were made using Nextclade and annotated using Figtree.

The HA, NA, and NS segments were amplified from all three samples. Precisely, 24.7% (19,584) of the 79,263 reads generated mapped to the three segments (Table 1). Mean depth ranged from 95× to 6,885×, and all sequences were >99% similar to the closest hits in GenBank (Table 1). Nextclade typed all three HA segments as B/Victoria, clade V1A.3a.2, and subclade C.5.1, while the NA segments belonged to clade B.7 (Table 1). Both HA and NA trees alongside BLASTn search results suggest that the three viruses might be part of two different transmission clusters (Fig. 1 and Table 1). None of the amino acid substitutions H134Y, H273N, or D197N associated with resistance (9) to NA inhibitors was found in any of the NAs described here.

**TABLE 1:** Details of sequences described in this study^*a*^

						Nextclade		GenBank		
S/N	Raw Reads (mean length)	Mapped reads	Segment (length)	Accession (GC%)	Mean depth	Clade	Subclade	Accession number	Percentage similarity (%)	Query cover (%)
1	37,142 (1,028)	684	HA (1,847)	PV221265 (43%)	191.9	V1A.3a.2	C.5.1	PV150802.1	99.89	100
			NA (1,527)	PV221266 (42%)	368.8	B.7		PV150820.1	100	100
			NS (1,066)	PV221267 (40%)	95.7			PV151094.1	100	100
2	14,127 (1,104)	2,116	HA (1,847)	PV221268 (42%)	468.7	V1A.3a.2	C.5.1	PP312032.1	99.57	100
			NA (1,529)	PV221269 (42%)	878.1	B.7		PV150836.1	99.87	100
			NS (1,066)	PV221270 (40%)	160.0			PV151094.1	99.81	100
3	27,994 (1,368)	16,784	HA (1,847)	PV221271 (43%)	4,357.9	V1A.3a.2	C.5.1	PV150802.1	99.89	100
			NA (1,527)	PV221272 (42%)	6,885.0	B.7		PV150820.1	100	100
			NS (1,066)	PV221273 (40%)	1,649.8			PV151094.1	100	100

^*a*^ Samples S/N 1, 2, and 3 were collected on the 10, 12, and 13 February 2025, respectively.

The IBV genomic segments described here belong to the clades documented to be circulating globally (10, 11). IBV vaccine strains selected for the 2025–2026 northern hemisphere influenza season have been shown to elicit an immune response capable of neutralizing viruses with HA from the clade (V1A.3a.2, subclade C.5.1) detected in this study (11).

## Data Availability

The reads and genomes described in this study have been deposited in the SRA and GenBank under accession numbers PRJNA1231480 (SRR32566846–SRR32566848) and PV221265–PV221273, respectively.
